# Application of thiourea ameliorates drought induced oxidative injury in *Linum usitatissimum* L. by regulating antioxidant defense machinery and nutrients absorption

**DOI:** 10.1016/j.heliyon.2024.e25510

**Published:** 2024-02-11

**Authors:** Khazra Fiaz, Muhammad Faisal Maqsood, Muhammad Shahbaz, Usman Zulfiqar, Nargis Naz, Abdel-Rhman Z. Gaafar, Arneeb Tariq, Fozia Farhat, Fasih Ullah Haider, Babar Shahzad

**Affiliations:** a Department of Botany, University of Agriculture, Faisalabad, Pakistan; b Institute of Grassland Science, Northeast Normal University, Key Laboratory of Vegetation Ecology, Ministry of Education, Jilin Songnen Grassland Ecosystem National Observation and Research Station, Changchun, 130024, China; c Department of Botany, The Islamia University of Bahawalpur, Bahawalpur, Pakistan; d Department of Agronomy, The Islamia University of Bahawalpur, Bahawalpur, Pakistan; e Department of Botany and Microbiology, College of Science, King Saud University, Riyadh 11451, Saudi Arabia; f Department of Botany, Government College Women University, Faisalabad, Pakistan; g Key Laboratory of Vegetation Restoration and Management of Degraded Ecosystems, South China Botanical Garden, Chinese Academy of Sciences, Guangzhou 510650, China; h University of Chinese Academy of Sciences, Beijing 100039, China; i Tasmanian Institute of Agriculture, University of Tasmania, Hobart, TAS 7001, Australia

**Keywords:** Drought, Flax, Metabolic adaptations, Photosynthesis, Sulfhydryl bioregulator

## Abstract

Thiourea (TU) is considered an essential and emerging biostimulant against the negative impacts of severe environmental stresses, including drought stress in plants. However, the knowledge about the foliar application of TU to mitigate drought stress in *Linum usitatissimum* L., has yet to be discovered. The present study was designed to assess the impact of foliar application of TU for its effects against drought stress in two flax cultivars. The study comprised two irrigation regimes [60% field capacity (FC) and the control (100% FC)], along with TU (0, 500, 1000 mg L^−1^) application at the vegetative stage. The findings indicated that drought stress reduced the shoot fresh weight (44.2%), shoot dry weight (67.5%), shoot length (41.5%), total chlorophyll (51.6%), and carotenoids (58.8%). Drought stress increased both cultivars' hydrogen peroxide (H_2_O_2_) and malondialdehyde (MDA). Foliar application of TU (1000 mg L^−1^) enhanced the growth and chlorophyll contents with or without drought stress. Under drought stress (60% FC), TU decreased MDA and H_2_O_2_ contents up to twofold. Moreover, TU application increased catalase (40%), peroxidase (13%), superoxide dismutase (30%), and total soluble protein contents (32.4%) differentially in both cultivars. Nevertheless, TU increased calcium (Ca^2+^) (42.8%), potassium (K^+^) (33.4%), and phosphorus (P) (72%) in shoots and decreased the elevated sodium (Na^+^) (28.2%) ions under drought stress. It is suggested that TU application (1000 mg L^−1^) enhances the growth potential of flax by enhancing photosynthetic pigment, nutrient uptake, and antioxidant enzymes under drought stress. Research outcomes, therefore, recommend that TU application can ameliorate drought-induced negative effects in *L. usitatissimum* L. seedlings, resulting in improved plant growth and mineral composition, as depicted by balanced primary and secondary metabolite accumulation.

## Graphical abstract

**Figure:**
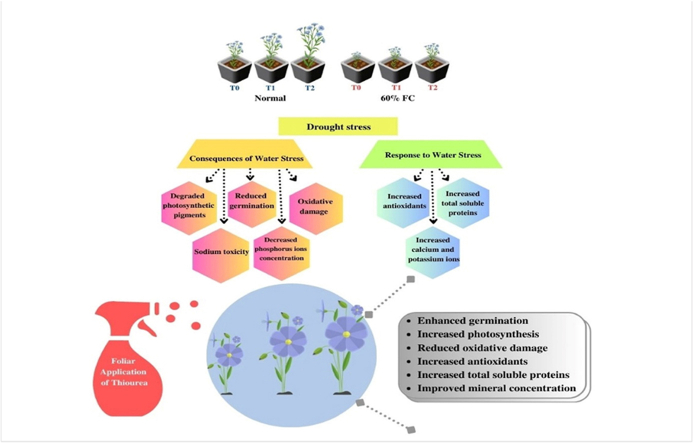

## 1 Introduction

Plants frequently encounter challenging environmental conditions, such as drought, which detrimentally affects their growth, biomass production, crop yield, and overall produce quality [1]. Among various environmental stresses, drought stress emerges as a significant constraint, leading to a substantial reduction in global crop productivity [2]. Projections indicate that by 2050, more than half of agricultural land will be impacted by drought, contributing to a 50% reduction in yield globally, particularly in regions prone to drought [3]. Currently, over 20% of the world's agricultural areas are grappling with drought conditions, resulting in a noteworthy decline in food production below the levels necessary to meet consumption demands [4]. The consequences of drought stress have already been speculated in various economically important crop species, such as hemp, ramie, flax, etc. [5]. Flax (*Linum usitatissimum* L.) is a healthy and prosperous source of bioactive compounds, including lignans, linolenic acid, omega-3 fatty acids, etc. [6]. Flax farming is expanding due to its seeds' excellent nutritious properties for humans and animal health [7,8]. However, in the previous two decades, flax demand has also increased tremendously as textile fibre [9]. Flax is susceptible to drought at all developmental stages [10]. Due to severe drought spells, there is an acute yield decline in flax production globally [11]. Drought stress caused a significant reduction in growth parameters, photosynthetic pigments, yield, and yield components in flax plants [12]. Still, the future climate change scenario is a severe challenge to cope with the production targets [13] that can restrict these plants' yield and nutritional properties [14].

Drought conditions are worsening globally because of climate change [15]. The drought stress conditions reduce cell division and elongation [16] and photosynthesis [17], which leads to low productivity. Drought stress reduced the yield in different crops, i.e., wheat (*Triticum aestivum* L.) (35.1%) [18], rice (*Oryza sativa* L.) (64%) [19], maize (*Zea mays* L.) (51.4%) [20], and barley (*Hordeum vulgare* L.) (20.3%) [21]. Drought also causes oxidative damage by elevated reactive oxygen species (ROS), such as H_2_O_2_ and MDA levels [22]. Plants stimulate their natural defense system comprising various antioxidants that balance the abiotic stresses induced by oxidative stress [23].

Researchers are continuously searching for sustainable crop production strategies under the threat of climate change-induced abiotic stresses [[24], [25], [26], [27]]. Among these, foliar applications of different natural and synthetic chemicals have been proposed to mitigate abiotic stresses-induced negative impacts on plants [28,29]. Exogenous application of synthetic chemicals such as TU enhanced growth and yield attributes of the plant due to the accumulation of total soluble proteins, sugars, osmolytes, and antioxidant enzyme activities under heat stress [30]. This synthetic plant growth regulator (PGR) is thiocarbamide, comprising 42% sulfur and 36% nitrogen, also known as TU [31]. TU is an organosulfur compound having structural similarity to urea; the oxygen (O) atom of urea is displaced by a sulfur (S) atom, and regardless of structural similarity, both compounds behave differently [32,33].

Moreover, TU has many beneficial effects, such as enhancing plant species' growth, physiology, and yield even when supposed to grow them in a stressed environment [[34], [35], [36]]. When applied exogenously, TU regulates vigorous seed sprouting and upgrades the gas exchange mechanism [30]. At physio-biochemical levels, it demonstrates an enhanced photosynthetic rate, nutrient uptake, and the rehabilitation of carbohydrate metabolism, subsequently improving protein biosynthesis [37]. TU upgraded necessary photosynthesis process and continuous flow of sugars from source to sink might work in better assimilation and translocation [38]. Recovery from stress involves reshuffling many metabolic pathways to compensate for drought-induced indemnities and reinstate plant growth and productivity [39]. Foliar application of TU improves plant germination and developmental processes, increasing the scavenging of excessive ROS such as MDA and H_2_O_2_ under drought [40]. TU decreases oxidative damage by upregulating non-enzymatic and enzymatic antioxidants [2] and increases photosynthesis by enhancing carotenoid and chlorophyll contents [41]. Furthermore, these compounds and their related derivatives are also used in the agrochemicals and entomology industry for better growth and development to create an eco-friendly and sustainable environment [42].

The application of TU via foliar spray has been acknowledged for its potential to mitigate drought stress in several crops such as rice [43], chickpea [44], wheat [45], and maize [46]. However, the exploration of TU's foliar application to alleviate abiotic stress in flax (*Linum usitatissimum* L.) remains an uncharted domain. Consequently, this experiment was conducted with the following objectives. Firstly, the study aimed to assess the efficacy of TU in enhancing the resilience of flax under drought-stress conditions. Secondly, it sought to identify flax varieties that demonstrate sensitivity or tolerance to drought stress through the application of TU. Lastly, the research aimed to delve into the physiological and biochemical mechanisms underlying the TU-induced enhancement of drought stress resistance in flax. The overarching hypothesis of this study posits that the application of TU contributes to the improvement of growth and development in flax under drought stress, mitigating the adverse effects of water deficiency through diverse physiological and biochemical mechanisms.

## 2 Materials and methods

### 2.1 Plant material and treatments

A completely randomized pot experiment having three biological replicates was performed at the Old Botanical Garden, University of Agriculture, Faisalabad, located at an altitude and longitude of 31.41, 73.07 respectively, to evaluate the growth regulating effect of TU on two flax varieties (Roshini and Chandni) under drought stress. The flax varieties were obtained from the Ayub Agricultural Research Institute (AARI) in Faisalabad, Pakistan. Pots measuring 26 cm × 23.5 cm × 22 cm were filled with 6 kg of soil. On November 28, 2021, ten seeds were sown in each pot and subsequently thinned to three plants per pot during the seedling stage. The soil used in this experiment was taken from Old Botanical Garden, UAF. It contains 1.07% organic matter, 1.31 and 1.10 ppm available phosphorus, and potassium with 51.07% saturation percentage, exhibiting sandy loam texture and 6.5, 4.55 mS pH and EC, respectively. The weather data for the experimental period is given in Fig. S1. The soil was kept moist at 95 ± 5% of the field water-holding capacity until the emergence of the main stem with many leaves (growth stage 5). Approximately 40 days after sowing (DAS), the plants underwent drought stress (60% FC), while a control group received no drought stress (100% FC). The 60% field capacity was determined by measuring the soil weight in the pots at both total water-holding capacity and complete dryness. Subsequently, the moisture content of a 100 g sample of oven-dried soil was calculated using a standard formula. The moisture levels of the drought-stressed plants were monitored by regularly assessing the soil in each pot, and adjustments were made by watering to maintain a moisture level corresponding to 60% of the calculated field capacity. This treatment persisted until harvesting to collect comprehensive data on growth and physio-biochemical attributes. Throughout the experiment, pots were weighed daily, and evapotranspiration water loss was controlled by adding water to maintain a specified weight for both the control (100% FC) and drought stress (60% FC) groups. Thiourea (CAS Number 62-56-6) was purchased from Sigma-Aldrich U.S.A. The experiment comprised three TU levels (T0: water spray/0 mg L^−1^, T1: 500 mg L^−1^, T2: 1000 mg L^−1^) applied after 14 d of drought application to both stressed (60% FC) and non-stressed (100% FC) plants. A 2000 mg L^−1^ stock solution of TU was prepared in distilled water and diluted accordingly. Each TU treatment was mixed with 1 mL of Tween-20 as a surfactant before foliar application. TU was applied once a week till the record of data.

### 2.2 Morphological attributes

Morphological attributes were recorded three weeks after TU application by uprooting plants from each treatment replicate. Shoot fresh weight (FW) and shoot dry weight (DW) were recorded using an electric balance (PA4102 Ohaus). For dry weights, shoot samples were oven-dried at 65 °C until they reached a constant dry weight. Shoot lengths were measured on a centimeter scale.

### 2.3 Photosynthetic pigment estimation

The leaf of each treatment was plucked and subjected to photosynthetic assessment [chlorophyll *a* (Chl a), chlorophyll *b* (Chl b), and carotenoids] using Arnon's method [47]. Freshly harvested sample leaves (0.1 g) were sliced into pieces and ground in 5 mL of 80% acetone (CAS No. 67-64-1) at 0 °C and kept overnight in the dark. The extracted leaves were centrifuged at 15,000 rpm for 5 min. Absorbance was recorded spectrophotometrically (IR-MECO U2020, Gmbh, Schwarzenbek, Germany) at 480, 645, and 663 nm.

### 2.4 Evaluation of enzymatic antioxidants

Fresh leaf samples (0.5 g) were thoroughly homogenized in 5 mL potassium phosphate buffer (pH 7.8) at 4 °C before centrifuging at 12,000 rpm for 15 min. The transparent layer was collected and stored in 500 μL aliquots at −20 °C for antioxidant estimations. The peroxidase (POD) activity was determined according to Chance and Maehly [48], a procedure using 100 μL tissue extract. The 3 mL reaction mixture containing 13 mM guaiacol (CAS No. 90-05-1), 5 mM H_2_O_2_ (CAS No. 7722-84-1), and 50 mM potassium phosphate buffer (pH 7.8), along with 100 μL enzyme assay. The gradual increase in optical density (OD) was recorded at 470 nm for 120s with a UV–visible spectrophotometer (IRMECO U2020, Gmbh, Schwarzenbek, Germany). The CAT activity was assessed following the methodology of Chance and Maely, [48]. The gradual decrease in absorbance at 290 nm was recorded for 120s. The increase and decrease in absorbance at 470 and 290 nm were expressed as Units mg^−1^ protein for POD and CAT, respectively. SOD activity was assayed following the method of Giannopolitis and Ries [49], by observing its ability to restrict the photochemical reduction of nitroblue tetrazolium (CAS No. 298-83-9) by 50%. The amount of total soluble proteins was determined at 595 nm using Bradford reagent [50], and the concentration was calculated from a standard curve prepared using bovine serum albumin.

### 2.5 Measurement of hydrogen peroxide (H_2_O_2_) and malondialdehyde (MDA)

H_2_O_2_ content was measured following Velikova et al. [51]. Briefly, foliage (0.5 g) was extracted with trichloroacetic acid (TCA, 0.1% w/v; CAS No. 76-03-9) before centrifuging at 12,000 g for 15 min and then potassium iodide (KI; CAS No. 7681-11-0), and potassium phosphate buffer (pH 7.0) were added to the supernatant. The OD was measured at 390 nm with a spectrophotometer. Heath and Packer's method [52] was used for MDA determination, which serves as an important indicator for membrane integrity under stress conditions. Approximately 500 mg leaf samples were homogenized completely with the addition of 3 mL TCA (5% w/v), and the crude extract was centrifuged (Model Camspec M330 UV/Vis) at 13,000 rpm for 15min at 4 °C. Further, a 20 % (w/v) TCA solution was mixed with 0.5% (w/v) thiobarbituric acid (TBA) with a final volume of 20 mL. The freshly prepared solution (4 mL) was thoroughly mixed with 1 mL supernatant in a clean glass test tube. Samples were incubated at 95 °C for 30 min. The reaction was terminated immediately at a freezing temperature. The samples were subjected to another round of centrifugation at 10,000 rpm for 10 min, and the OD was recorded at 532 nm with a spectrophotometer, corrected for non-specific turbidity by subtracting the absorbance at 600 nm.

### 2.6 Plant nutrients analysis

The plants were uprooted from each treatment and replicated, and their shoots were oven-dried for 72 h, till the observance of constant dry weight. Each oven-dried (65 °C/72 h) shoot sample (100 mg) was ground into a coarse powder and digested in an acid mixture having HNO_3_:HClO_4_ (5:1 v/v). After 3 h of incubation at 30 °C, samples were heated on a hot plate at 370 °C, before adding H_2_O_2_ until transparent (tinted yellow) condition. Afterwards, it was filtered and diluted by adding up to 50 mL of distilled H_2_O [53]. A flame photometer (Sherwood flame Photometer-410, Sherwood Scientific Ltd. Cambridge, UK) was used to determine potassium (K^+^), sodium (Na^+^), and calcium (Ca^2+^) contents. Phosphorus (P) contents were analyzed in Barton reagent using a spectrophotometer.

### 2.7 Statistical analysis

The experiment was set up in a three-factor arrangement with a completely randomized design and three replicates per treatment. The ANOVA of all parameters was undertaken using the statistics software CoStat. A correlation matrix between the treatments and cultivars was used to assess the relationship between different attributes using R Core Team 2019 (R4.3.1 statistical software).

## 3 Results

### 3.1 TU improves flax growth under drought stress

**Fig. 1:**
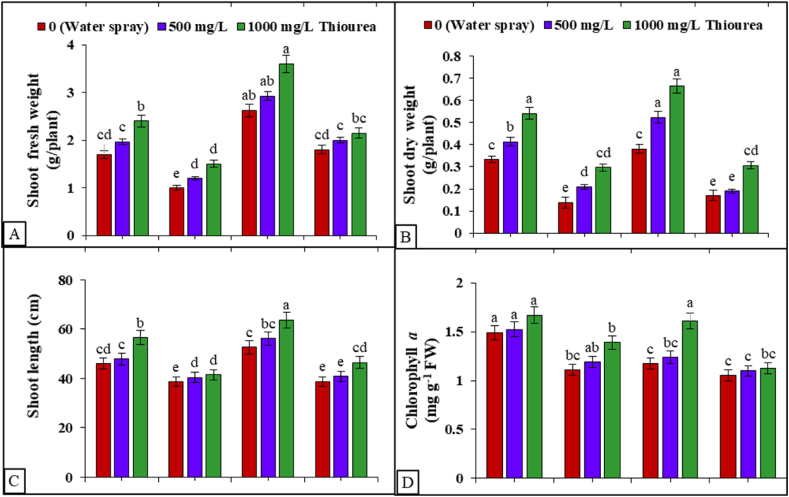
Exogenous application of TU modulates (A) shoot fresh weight (B) shoot dry weight (C) shoot length (D) chlorophyll *a* content in shoots of *L. usitatissimum* under drought stress compared to well-watered plants. The presented data represent the mean value along with the standard deviation (SD) obtained from three biological replicates.

Under the water deficit condition (60%FC), both flax cultivars showed (P ≤ 0.001) decrease in shoot lengths and shoot FW and DW. However, drought-stressed plants with TU application increased total FW and DW, but the values were still low compared to the control plants (Table S1 suppl). The morphological data depicted that cvs. Chandni performed better than cvs. Roshini under water-stressed and normal conditions (Fig. 1A–C). TU application at 1000 mg L^−1^ substantially increased shoot FW and DW relative to the control. The experimental findings suggested that TU (1000 mg L^−1^) application enhanced SFW (33%), SDW (58%) in both flax cultivars but showed a non-significant response to TU under drought stress (Fig. 1A–C). During the experimental period of TU foliar treatments, the interactive effect of cultivar × TU levels × drought stress was found to be non-significant. Notably, 1000 mg L^−1^ of TU emerged as the most effective concentration in mitigating drought stress for both flax cultivars (Fig. 1A–C).

### 3.2 TU improves photosynthetic pigments of flax cultivars under drought conditions

**Fig. 2:**
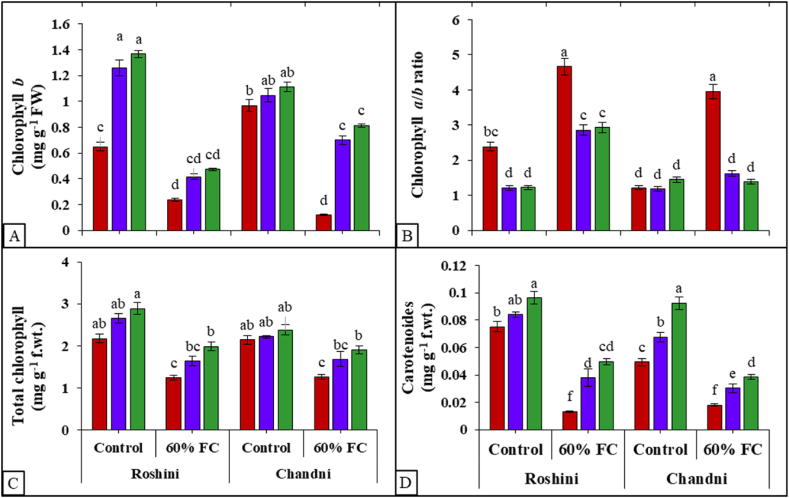
Exogenous application of TU modulates (A) chlorophyll *b* content, (B) chlorophyll *a*/*b* ratio, (C) total chlorophyll content, and (D) carotenoid content in shoots of *L. usitatissimum* under drought stress compared to well-watered plants. The presented data represent the mean value along with the standard deviation (SD) obtained from three biological replicates.

With drought stress, TU foliar application inhibited the decreasing trend in chlorophyll (Chl) contents of both flax cultivars. However, drought stress alone (P ≤ 0.001) decreased Chl a (42%), Chl b (54%), and total Chl contents (49%) in both cultivars, more so without TU application (Table S1 suppl, Figs. 1D, 2A-D). The application of TU at a concentration of 1000 mg L^−1^ demonstrated remarkable efficacy in alleviating the detrimental impacts of drought, leading to a substantial increase in Chl a, Chl b, and total Chl contents, with the exception of the Chl a/b ratio across both cultivars. TU application (1000 mg L^−1^) enhanced Chl a (15%), Chl b (38%), and total Chl (19%) contents under control and water-stressed conditions (Table S1 suppl, Fig. 2A–D) relative to no TU application. The Chla/b ratio considerably decreased in both cvs. with TU application, except in cv Chandni under drought stress. Roshini, under drought stress, had the highest Chl a/b ratio, whereas Chandni, under control conditions, had the lowest. Total Chl contents increased with TU application in both cvs. under normal and drought conditions. Drought stress decreased (P ≤ 0.001) carotenoid content (87–90%) in both cvs. (Fig. 2D), compared to control plants, while TU application increased carotenoid content (77–80%) compared to the water spray flax plants with a limited supply of water. Roshini had the highest carotenoid content with TU application with and without drought stress (Fig. 2D). Overall, TU application exhibited positive interaction with cultivars under drought stress with respect to Chl a, Chl b and Chl a/b (Table S1 suppl). The higher TU level (1000 mg L^−1^) was more effective than the lower level (500 mg L^−1^) at combating the adverse effects of drought, particularly in Roshini (Table S1 suppl, Figs. 1D, 2A–D).

### 3.3 TU improves antioxidant pool with concurrent decrease of oxidant species of flax cultivars under drought stress

**Fig. 3:**
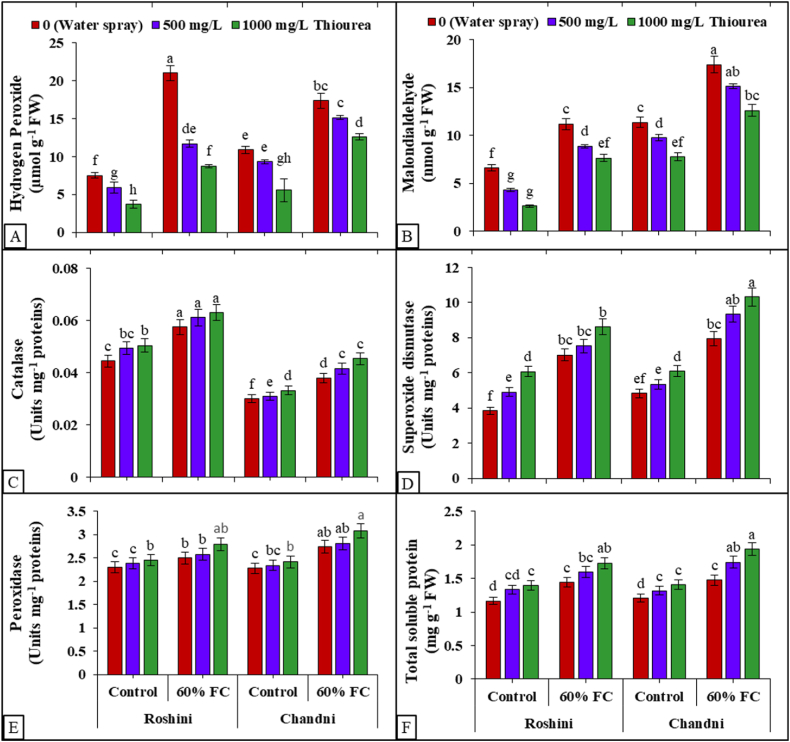
Exogenous application of TU modulates (A) hydrogen peroxide (H_2_O_2_) level, (B) malondialdehyde (MDA) content, (C) catalase (CAT) activity, (D) superoxide dismutase (SOD) activity, (E) peroxidase (POD) activity, and (F) total soluble protein (TSP) content in shoots of *L. usitatissimum* under drought stress compared to well-watered plants. The presented data represent the mean value along with the standard deviation (SD) obtained from three biological replicates.

Drought stress elevated H_2_O_2_ levels in both cultivars, with a more pronounced effect observed in Roshini compared to Chandni (Table S1 suppl, Fig. 3A). Conversely, foliar application of TU resulted in a reduction of H_2_O_2_ levels in both cultivars, with a more significant impact noted at the higher application rate (1000 mg L^−1^) compared to the lower application rate (500 mg L^−1^). Moreover, the foliar application of TU resulted in a significant reduction of H_2_O_2_ contents by 66% when compared to plants treated with a water spray under 60% FC conditions (Fig. 3A). Roshini cultivar was more sensitive to drought stress than Chandni by accumulating more H_2_O_2_ contents without TU application. Drought stress also influenced the MDA contents in both cultivars of flax (Table S1 suppl). Foliar application of 1000 mg L^−1^ TU decreased MDA contents relative to 500 mg L^−1^ TU and the control (0 mg L^−1^ TU). Regarding MDA contents, the cvs. Chandni exhibited a higher accumulation of MDA compared to the cvs. Roshani. Hence, Roshani was better adapted to drought stress than Chandni with respect to MDA to ensure better survival under changing environmental constraints (Fig. 3B). Drought stress enhanced the antioxidant activities in both flax cvs., more so when combined with 1000 mg L^−1^ TU (Fig. 3C–F). Foliar TU application enhanced SOD, CAT, and POD activities in both cvs. With and without drought stress, more so in Roshini than in Chandni (Fig. 3C–E). TU application enhanced SOD (42%), CAT (33%) and POD (7%) under a limited supply of water compared to fully irrigated flax plants (Fig. 3).

### 3.4 TU improves the nutrient profile of flax cultivars under drought stress

**Fig. 4:**
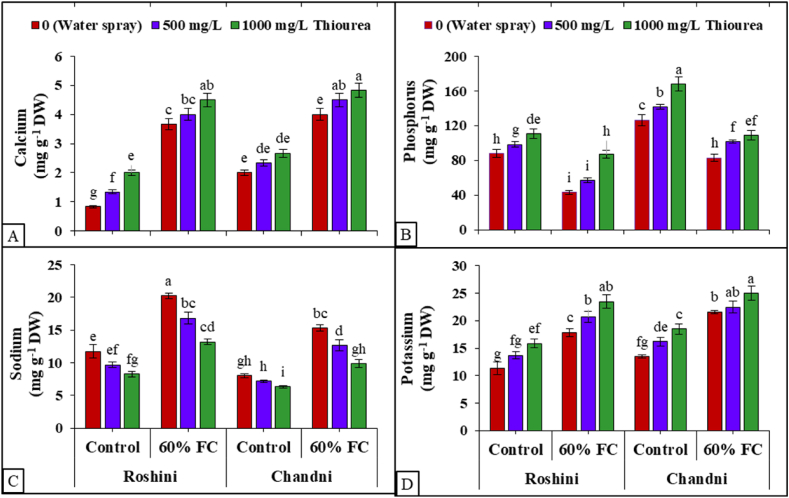
Exogenous application of TU modulates (A) calcium (Ca^2+^) content, (B) phosphorus (P) content, (C) sodium (Na^+^) content, and (D) potassium (K^+^) content in shoots of *L. usitatissimum* under drought stress compared to well-watered plants. The presented data represent the mean value along with the standard deviation (SD) obtained from three biological replicates.

Foliar TU application changed the nutrient profile of both flax cultivars under drought stress, increasing Na^+^, Ca^2+^, and K+ contents and decreasing P content relative to normal conditions (100% FC) (Fig. 4A). In contrast, foliar TU increased element concentration in both flax cultivars under normal and drought-stressed conditions (Fig. 4B). The increase in Na^+^ decreased with increasing TU concentration in both cultivars (Fig. 4C). Under control conditions, Roshini exhibited the lowest shoot Ca^2+^ and K^+^ contents (Fig. 4A and D). Overall, Chandni had a better nutrient profile than Roshini.

### 3.5 Growth, physiological, and biochemical attributes

**Fig. 5:**
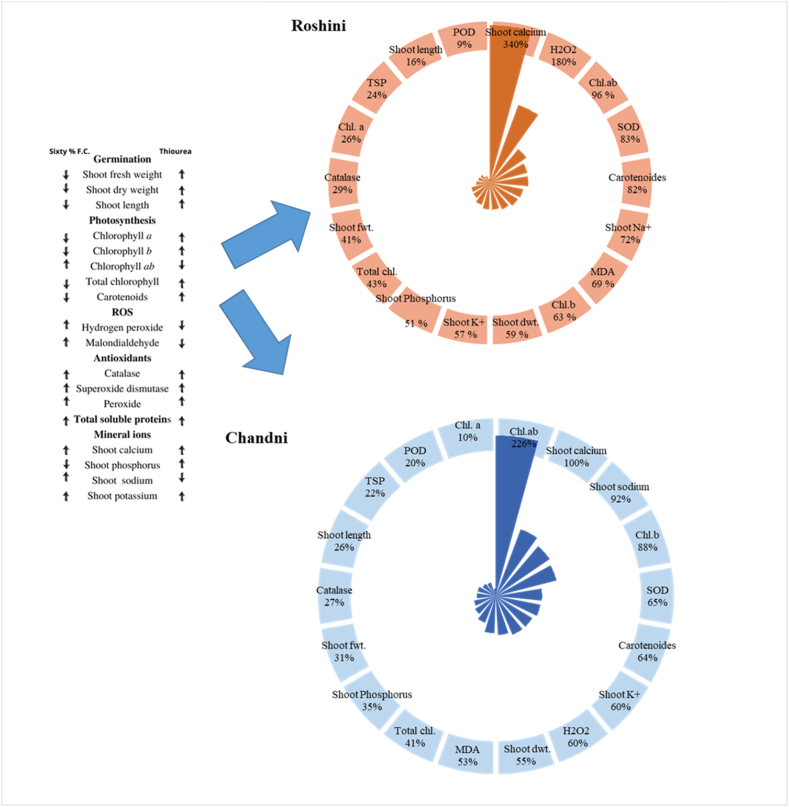
Donut Wind Rose diagram showing the percentage increase or decrease in plant attributes of two *Linum usitatissimum* L. cultivars (Roshini and Chandni) under drought stress with TU application.

**Fig. 6:**
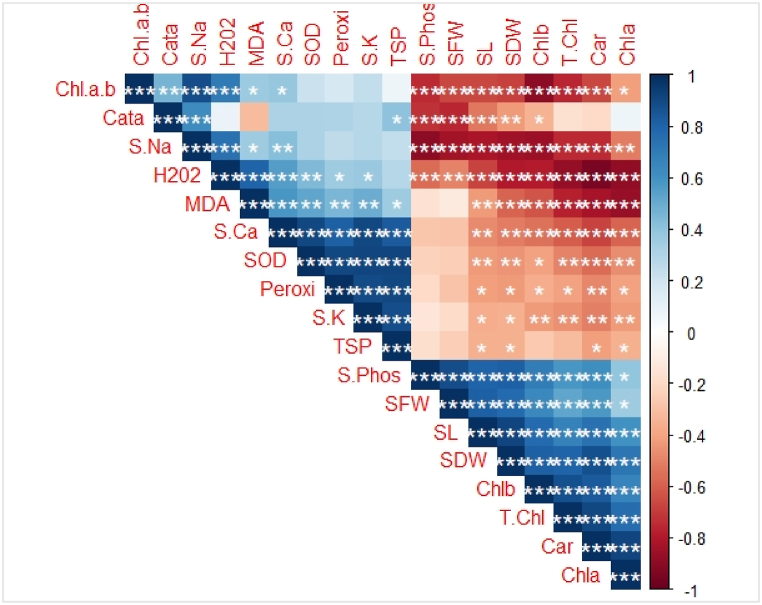
Correlation among Chl. a.b (chlorophyll *a*/b ratio), Cata (catalase), S.Na (shoot sodium), H_2_O_2_ (hydrogen peroxide), MDA (malondialdehyde), S.Ca (shoot calcium), SOD (Superoxide dis-mutase), Peroxi (peroxidase), S. K (shoot potassium), TSP (total soluble proteins), S. Phos (shoot phosphorus), SFW (shoot fresh weight), SL (shoot length), SDW (shoot dry weight), Chl b (chlorophyll *b*), T. Chl (total chlorophyll), Car (carotenoids), Chla (chlorophyll *a*).

A donut wind rose diagram was used to evaluate the dynamics of various traits under water deficit and TU application (Fig. 5). The percentage increase (↑) and decrease (↓) are shown for different attributes (Fig. 5). Carotenoid content (33%) and Chl b content (55%) increased the most in Roshani and Chandni, respectively, under combined drought stress and TU application (Fig. 6). Pearson's correlation revealed positive and negative correlations between growth and yield characteristics and various physio-biochemical parameters, antioxidative metabolism, and nutrient attributes (Fig. 6). Significant positive correlations occurred between shoot Na^+^ content and the Chl a/b ratio and CAT activity, H_2_O_2_ level and the Chl a/b ratio, shoot Na^+^ content, and MDA content, SOD activity and shoot Ca^2+^ content, POD activity and shoot Ca^2+^ content and SOD activity, shoot K^+^ content and POD activity and shoot Ca^2+^ content, TSP and shoot Ca^2+^ and K+ contents and SOD and POD activities, shoot FW and shoot P content, shoot length and shoot P content and shoot FW, shoot DW and shoot P content, shoot FW, and shoot length, Chl b content and shoot P content, shoot FW, shoot DW and shoot length, Total Chl and shoot P content, shoot FW, shoot length, shoot DW, and Chl b, carotenoid content and shoot P content, shoot FW, shoot DW, shoot length, Chl b, and total Chl, Chl a and shoot DW, shoot length, Chl b, and total Chl (Fig. 6).

Significant negative correlations occurred between shoot P contents and the Chl a/b ratio, CAT activity, and shoot Na^+^ content, shoot FW and Chl a/b ratio, CAT activity, and shoot Na^+^ content, shoot length and shoot Na^+^ content, shoot DW and shoot Na^+^ content, Chl b and the Chl a/b ratio, shoot Na^+^ content, and H_2_O_2_ level, total Chl and the Chl a/b ratio, shoot Na^+^ content, H_2_O_2_ level, and MDA content, carotenoid content and the Chl a/b ratio, shoot Na^+^ content, H_2_O_2_ level, and MDA content, Chl b and H_2_O_2_ level and MDA content (Fig. 6).

## 4 Discussion

Drought conditions are prevailing around the globe very rapidly due to climate-induced variations in precipitation and competing land interests for economic growth [54], putting global food security at risk [31]. It is widely recognized that water shortage significantly influences various metabolic and physiological disorders in plants [55,56]. We observed that both cultivars of flax showed a decline in shoot length, shoot fresh and dry weight in response to drought treatments (Fig. 1A–C), which was validated via earlier cited literature [57]. Turgor pressure decreases at the onset of drought stress, inhibiting cell proliferation and development [58]. Drought stress impairs photosynthesis (Figs. 1D, 2A–D), thus negatively affecting plant growth [59]. Tolerance to abiotic stresses in plants is a complex phenomenon, and TU may modulate several underlying mechanisms involved in the growth and development of plants [60]. TU is a synthetically derived plant growth-promoting molecule acquiring nitrogen and sulfur in a proportion of 36:42% that has an enormous capacity to exhibit plant stress tolerance or mitigation [31]. It has a wide range of biological actions in agricultural plants [61]. The current study also advocates the positivity of TU under drought stress by enhancing the growth and photosynthetic features of both cultivars of flax. It has been established that, TU application is significantly involved in the enhancement of cell division at different phases of cellular growth [62]. Foliar TU application improved plant vigor in two bread wheat cultivars by mitigating the adverse effects of drought [63]. Similarly, foliar TU application increased shoot FW and DW in *Coriandrum sativum* L. under drought stress [64]. In cluster beans (Cyamopsis tetragonoloba L.), TU application upregulated growth by enhancing nitrate reductase activity and increasing the photosynthetic process under a limited supply of water [65]. In onion (*Allium cepa* L.), TU improved and elevated numerous metabolic processes to induce drought tolerance [66].

**Fig. 7:**
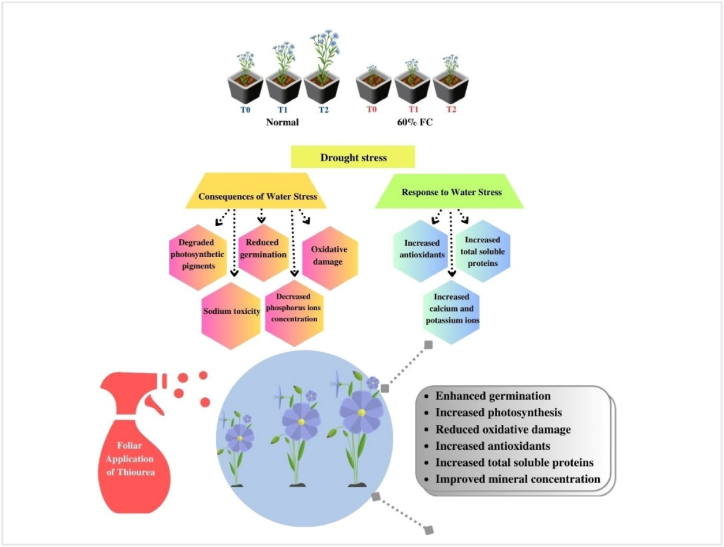
The schematic diagram exhibiting the beneficial role of foliar Thiourea application against drought-induced phytotoxicity in two *Linum usitatissimum* L. cultivars.

Drought stress has the potential to induce chloroplast deterioration through chlorophyll oxidation and degradation ([67]; Fig. 7). It is likely to affect chlorophyll biosynthesis, which correlates positively with stomatal conductance and photosystem II. Drought stress promotes the generation of stress signal molecules such as H_2_O_2_ and O_2_^●–^, pivotal indicators for chlorophyll loss and lipid peroxidation [68]. The restricted water supply reduces the photosynthetic mechanism and source strength. Furthermore, drought stress leads to decreased turgor pressure in phloem cells, enhancing the viscosity of sucrose syrup and impeding its flow via the sieve tubes and companion cells toward the sinks (seeds), consequently affecting seed vigor [68]. Drought stress also impacts ribulose bisphosphate carboxylase (RuBisCo) and other photosynthetic-related enzymes, contributing to the deterioration of photosynthetic pigments [23]. TU-mediated improvements in photosynthetic pigments may be attributed to its positive effects on rubisco activity, enhancing both photosynthetic rates [65] and the respiratory capacity of roots [31]. Exogenous treatment of TU considerably enhances the photosynthetic pigments of maize seedlings, concurrently increasing cell metabolic rates and delaying plant senescence by protecting chloroplasts from senescing [61]. Under stress conditions, TU increases Chl a and Chl b contents while decreasing the Chl a/b ratio, compensating for plant metabolic processes [69,70].

Furthermore, it is anticipated that TU, a ROS scavenger, have divergent impacts on the cellular redox state. This state is defined as a comprehensive ratio of the reduced to oxidized forms of all redox couples within the cell. At the organismic level, the regulation of the redox state is governed by enzymes that scavenge or produce ROS and metabolites that possess antioxidant properties ([71]; Fig. 7). The synthesis of ROS induced by stress is regarded as a crucial process in initiating a series of adaptive responses, such as early stomatal closure [72,73], regulation of xylem Na^+^ loading [74,75], and Na^+^ compartmentalization [76]. Concurrently, increased ROS generation in saline circumstances may have unfavorable effects on intracellular ionic balance. Protease and endonuclease activity, triggered by ROS-induced cytosolic K^+^ depletion [77,78], leads to programmed cell death. Therefore, maintaining stable concentrations of ROS is essential to support redox biology and actively participate in signalling pathways [79,80]. In the current study, ROS and lipid peroxidation peaked in both flax cultivars under drought stress (Fig. 2). Lee et al. [81] have clearly demonstrated a negative correlation between the decrease in dry biomass of plants to enhanced production of oxidant species and lipid peroxidation in white clover leaves under drought stress conditions. The non-toxic accumulation of ROS-activated Ca^2+^-permeability which means that Ca^2+^-activated NADPH oxidases work together with Ca^2+^-activated NADPH oxidases to make and boost stress-induced Ca^2+^ and ROS signals [82]. On the other hand, oxidant species break the ester bonds between glycerol and fatty acid molecules to deteriorate membrane integrity, causing an oxidative burst in the cell [69,81]. However, Kaya et al. [83] reported that drought stress combined with TU application promoted maize growth indices by reducing membrane permeability (MP), MDA, and H_2_O_2_ levels, modifying the antioxidant pool, and enhancing photosynthetic pigments [41]. Foliar TU application also improved flax germination by reducing H_2_O_2_ and MDA levels [84]. TU could be involved in the recovery mechanism of fatty acid chains [60], as a ROS-scavenger and an antioxidant defender [85]. The present study demonstrated that drought stress stimulated the antioxidant defense response of both flax cultivars, further strengthened by foliar TU application (Fig. 2), which is also according to the findings of Mohammadi et al. [86] on chickpeas. Spinach (*Spinacea oleracea* L.) under various levels of drought stress (100, 80, 60, and 40% FC) revealed that 40 % FC improved CAT and POD activities [28]. Enhancing the activities of antioxidants could minimize or avoid oxidative damage caused by drought stress, thus increasing plant's tolerance to water scarcity [87]. Foliar TU application enhanced CAT and SOD activities in sunflowers (*Helianthus annus* L.) exposed to limited water supply [88].

A sufficient mineral nutrient supply in the rhizosphere is essential for plants to show avoidance mechanisms or mitigation responses under environmental stresses [87]. Reduced Ca^2+^ uptake in stressful situations led to less Ca^2+^ sticking to the plasma membrane [89,90]. This caused a change in membrane permeability, which could be seen as K^+^ leaking out of the cell [91]. TU demonstrates the capacity to modulate nutrient status by enhancing nutrient uptake translocation and regulating essential plant metabolic pathways, thereby improving stress tolerance [31]. In the current study, drought stress led to an increase in Ca^2+^, K^+^, and Na^+^ contents but a decrease in shoot P content (Fig. 3). Similar nutrient profile changes were observed in castor beans (*Ricinus communis* L.) under various drought levels [92]. Similarly, Ge et al. [93] reported a decrease in P uptake in maize under drought stress. The uptake of Ca^2+^, K^+^, and Na^+^ may be associated with osmotic adjustment, maintaining homeostasis in plants particularly susceptible to environmental stresses [94]. Warren et al. [95] found a positive correlation between transpiration rate and nutrient absorption, especially P, in plants. In the current study, TU demonstrated an improvement in mineral nutrient status (Ca^2+^, K^+^, and P) and reduced Na^+^ toxicity in both flax cultivars. Similarly, in maize, TU enhanced water status by increasing mineral concentrations in the plant [31]. The foliar spray of TU has been associated with an enhancement in the nutritional quality of plants [96]. These findings align with research indicating that TU regulates N and P metabolism, increases photosynthesis, and results in higher quantities of starch, soluble proteins, and lipids [97].

## 5 Conclusions

This study showed that foliar TU application to *Linum usitatissimum* L. could be a practical solution for minimizing the adverse effects of drought. Foliar TU application improved nutrient uptake under drought stress, balancing the growth, photosynthetic and antioxidant response. In both drought stress and control conditions, the application of TU resulted in enhanced growth attributes and increased levels of photosynthetic pigments, antioxidants, and mineral nutrients while concurrently downregulating ROS. In both cultivars, 1000 mg L^−1^ TU was more efficient than 500 mg L^−1^ TU at mitigating drought stress. The application of vitamin thiamine provides a valuable resource for comprehending various aspects of primary and secondary metabolism in plants. The exogenous application of thiamine presents a promising opportunity to enhance abiotic resistance mechanisms, particularly in mitigating the effects of drought stress. This approach has the potential to alleviate the economic pressure of low productivity and address severe malnourishment issues in the face of climate variability. In summary, this study provides valuable insights for researchers aiming to unravel the physiological mechanisms behind TU-induced drought stress tolerance in flax seedlings. These findings can serve as a roadmap for future investigations focusing on cellular-level understanding in this context.

## Additional information

No additional information is available for this paper.

## Data availability statement

We have already shared all data in this manuscript.

## CRediT authorship contribution statement

**Khazra Fiaz:** Writing – original draft, Data curation, Conceptualization. **Muhammad Faisal Maqsood:** Formal analysis. **Muhammad Shahbaz:** Data curation, Conceptualization. **Usman Zulfiqar:** Supervision, Investigation. **Nargis Naz:** Formal analysis. **Abdel-Rhman Z. Gaafar:** Writing – review & editing, Resources. **Arneeb Tariq:** Writing – original draft. **Fozia Farhat:** Writing – original draft, Validation. **Fasih Ullah Haider:** Writing – review & editing, Supervision. **Babar Shahzad:** Supervision.

## Declaration of competing interest

The authors declare that they have no known competing financial interests or personal relationships that could have appeared to influence the work reported in this paper.
