# Supplementary material for: Application of thiourea ameliorates drought induced oxidative injury in Linum usitatissimum L. by regulating antioxidant defense machinery and nutrients absorption
Source: Heliyon. 2024 Feb 11;10(4):e25510. doi: 10.1016/j.heliyon.2024.e25510 (PMC10881316; doi:10.1016/j.heliyon.2024.e25510)
Supplement: Multimedia component 1 [file mmc1.docx]

**
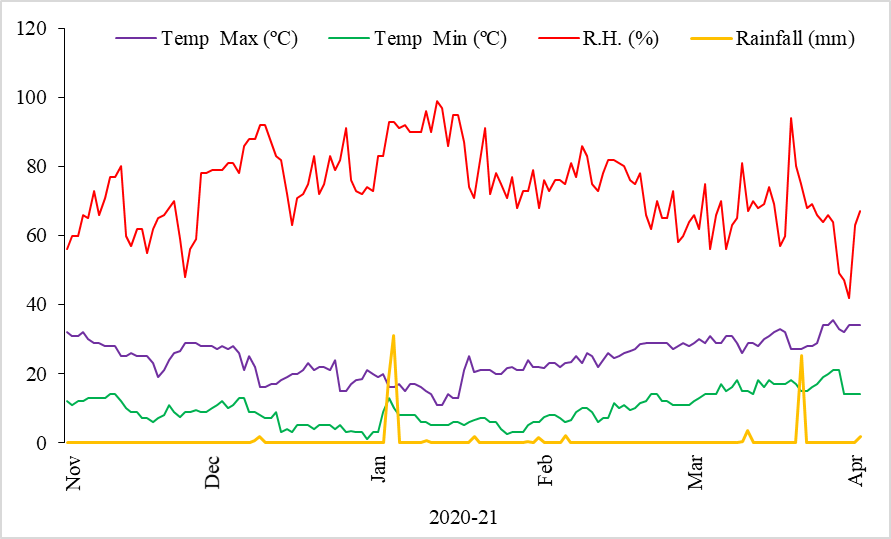
**

**Figure 1**: Metrological data of experimental period (2020–2021) (Source: https://en.climate-data.org/asia/pakistan/punjab/faisalabad-764568/)
