# Supplementary material for: Application of thiourea ameliorates drought induced oxidative injury in Linum usitatissimum L. by regulating antioxidant defense machinery and nutrients absorption
Source: Heliyon. 2024 Feb 11;10(4):e25510. doi: 10.1016/j.heliyon.2024.e25510 (PMC10881316; doi:10.1016/j.heliyon.2024.e25510)
Supplement: Multimedia component 2 [file mmc2.docx]

**Suppl. Table 1**. Means of sums of square of different growth, physiological and biochemical attributes of *L. usitatissimum* L. with thiourea (TU) and drought

| SOV | df | Shoot FW | Shoot DW | Shoot length | *Chl a* | *Chlb* | *Chla/b* |
| --- | --- | --- | --- | --- | --- | --- | --- |
| Cultivar (CV) | 1 | 7.111*** | 0.023** | 190.44** | 0.291*** | 0.031* | 4.327*** |
| Drought (D) | 1 | 7.747*** | 0.598*** | 1465.614*** | 0.762*** | 3.310*** | 20.079*** |
| Thiourea (TU) | 2 | 1.217*** | 0.118*** | 208.564*** | 0.192*** | 0.679*** | 5.992*** |
| CV× D | 1 | 0.174ns | 0.017* | 66.967* | 0.016ns | 0.107*** | 1.908*** |
| CV× TU | 2 | 0.0031ns | 0.0006ns | 5.3164ns | 0.0006ns | 0.003ns | 0.024ns |
| D × TU | 2 | 0.143ns | 0.008ns | 27.188ns | 0.019* | 0.001ns | 2.904*** |
| CV× D × TU | 2 | 0.042ns | 0.003ns | 6.773ns | 0.049*** | 0.245*** | 0.749** |
| Error | 24 | 0.082 | 0.002 | 14.104 | 0.004 | 0.006 | 0.087 |
| SOV | **df** | **Total chl** | **Caroten.** | **H_2_O_2_** | **MDA** | **CAT** | **SOD** |
| Cultivar (CV) | 1 | 0.250* | 0.001*** | 37.870*** | 268.595*** | 0.003*** | 7.748*** |
| Drought (D) | 1 | 5.621*** | 0.019*** | 475.016*** | 230.806*** | 0.001*** | 92.822*** |
| Thiourea (TU) | 2 | 1.030*** | 0.002*** | 128.914*** | 47.334*** | 0.0001*** | 9.568*** |
| CV× D | 1 | 0.227* | 0.0002* | 6.357* | 1.096ns | 0.00002ns | 2.538* |
| CV× TU | 2 | 0.064ns | 0.00001ns | 10.919** | 0.471ns | 0.000001ns | 0.076ns |
| D × TU | 2 | 0.034ns | 0.00005ns | 16.880*** | 0.126ns | 0.000001ns | 0.100ns |
| CV× D × TU | 2 | 0.040ns | 0.00026** | 16.690*** | 0.518ns | 0.00001ns | 0.447ns |
| Error | 24 | 0.033 | 0.00003 | 1.482 | 0.677 | 0.000009 | 0.345 |
| SOV | **df** | **POD** | **TSP** | **Shoot Ca^2+^** | **Shoot P** | **Shoot Na^+^** | **Shoot K^+^** |
| Cultivar (CV) | 1 | 0.115*** | 0.043** | 4.083*** | 15064.862*** | 98.163*** | 49.730*** |
| Drought (D) | 1 | 1.323*** | 1.064*** | 47.430*** | 16031.85*** | 353.25*** | 420.083*** |
| Thiourea (TU) | 2 | 0.161*** | 0.247*** | 1.756** | 3371.758*** | 50.739*** | 58.690*** |
| CV× D | 1 | 0.175*** | 0.030* | 0.75ns | 274.432ns | 5.672* | 0.002ns |
| CV× TU | 2 | 0.0008ns | 0.004ns | 0.074ns | 21.800ns | 1.481ns | 0.545ns |
| D × TU | 2 | 0.029* | 0.021* | 0.032ns | 10.427ns | 13.076*** | 0.137ns |
| CV× D × TU | 2 | 0.002ns | 0.009ns | 0.065ns | 337.578* | 0.0201ns | 1.754ns |
| Error | 24 | 0.008 | 0.003 | 0.207 | 98.952 | 1.313 | 1.740 |

SOV: source of variation, df: degrees of freedom, DW: dry weight, FW: fresh weight, Chl: chlorophyll, Caroten.: carotenoids, H_2_O_2_: hydrogen peroxide, MDA: Malondialdehyde, CAT: catalase, SOD: Superoxide dismutase, POD: peroxidase, TSP: total soluble proteins. ***, **,* highly significant, significant, least significant at 0.001, 0.03 and 0.05 respectively, while ‘**ns’** for non-significant
